# Effect of the Sodium Polyacrylate on the Magnetite Nanoparticles Produced by Green Chemistry Routes: Applicability in Forward Osmosis

**DOI:** 10.3390/nano8070470

**Published:** 2018-06-27

**Authors:** Juan Zufía-Rivas, Puerto Morales, Sabino Veintemillas-Verdaguer

**Affiliations:** Instituto de Ciencia de Materiales de Madrid, ICMM/CSIC Sor Juana Inés de la Cruz 3, Cantoblanco, 28049 Madrid, Spain; juanzufiar@gmail.com (J.Z.-R.); puerto@icmm.csic.es (P.M.)

**Keywords:** magnetic nanoparticles, sodium polyacrylate, nanocomposites, draw solutes, forward osmosis

## Abstract

Aqueous dispersions of magnetic nanocomposites have been proposed as draw electrolytes in forward osmosis. One possible approach for the production of nanocomposites based on magnetite nanoparticles and sodium polyacrylate is the synthesis of the magnetic iron oxide by coprecipitation or oxidative precipitation in the presence of an excess of the polymer. In this work, we explored the effect of the polymer proportion on the nanomaterials produced by these procedures. The materials obtained were compared with those obtained by the coating of magnetite nanocrystals produced beforehand with the same polymer. The samples were characterized by chemical analysis, photon correlation spectroscopy, thermogravimetry, X-ray diffraction, infrared spectroscopy, transmission electron microscopy, and magnetometry. The general trend observed is that the polymers heavily modify the texture of the magnetic material during the synthesis, with a drastic reduction of the particle size and magnetic response. The polycrystalline texture that is generated permits the incorporation of the polymer both on the external surface and in the intergranular space. The aqueous dispersions of the nanocomposites were highly stable, with a hydrodynamic size that was roughly independent of the polymer/magnetite ratio. Such dispersions show an osmotic pressure that is proportional to the concentration of the polymer. Interestingly, the proportionality constant was similar to that of the free polymer only in the case of the samples prepared by oxidative precipitation, being lower in the case of the samples prepared by coprecipitation. Finally, the possibilities of using these materials as draw electrolytes in forward osmosis will be briefly discussed.

## 1. Introduction

Forward osmosis (FO) is considered a technology for water desalination and the reuse of wastewater with great potential [1,2,3,4,5]. The forward osmosis process uses an electrolyte of high osmotic pressure (draw electrolyte) to drive the water molecules through a membrane from the waste or seawater instead of the high pressure pumps used in the standard reverse osmosis (RO). The benefits of such a process, apart from the pressure reduction that operates on the membrane [6], come from the reduction of the energetic cost of the water permeation through the membrane. However, the reduction in cost of the overall FO process is highly dependent on the water flux and the recover feasibility of the draw solute from the permeated water [7]. In spite of the high economic cost, simple inorganic salts and water distillation for recovering were initially explored [8]; phosphates and organic molecules were also proposed due to their low reverse flux through the membrane [9,10,11,12]. Considering microfiltration as the separation technique for recovering the solute with negligible reverse flux, the sodium salts of hydrophilic polymers such as polyacrylic acid (PAA) [13] polyaspartic acid [14], poly 4-styrenesulphonic acid [15], ethylenediaminetetra-acetic acid (EDTA) [16], and dendrimers [17] have been tested. Thermoresponsive substances that are able to produce sols of high osmotic pressure and split in two phases with small changes in temperature could be used as draw solutes. In this case, the water was advantageously separated from the water-rich phase. These substances could be polymers [18,19], ionic liquids [20], and microgels [21,22,23]. Functional nanoparticles that combine several functionalities are the subject of active research in Materials Science [24,25]. Examples of such systems that are employed in the preparation of draw electrolytes are carboxylate-functionalized carbon dots that produce high osmotic pressure and could be combined with microfiltration to recuperate the water [26], and magnetic nanoparticles (MNPs) that appear to be ideal, given their recover facility from the diluted draw solution by means of a magnetic field. Aqueous dispersions of bare MNPs have negligible osmotic pressure, even at the maximum concentration attainable in any FO plant (roughly 50 wt %), and need to be functionalized with molecules that generate it. The functionalization molecules are often polyelectrolytes, as PAA [27,28], dextran [29], chitosan [30], copolymers polyethylene oxide–polyacrylic acid [31], and silanes [32]. Really good performances were obtained by coating with hydrophilic thermoresponsive polymers. These polymers, in addition to the creation of osmotic pressure, facilitate the magnetic separation by changing the polymer conformation [33,34]. In recent reviews, the topic of the diverse draw solutes of high osmotic pressures that have been claimed to be good for FO were fully addressed [35,36]. In this work, we will focus on the attainment of high magnetic response in order to make the recovering and recycling of the MNPs easier; this will undoubtedly lead to a detriment of the osmotic pressure [35]. The objective is to determine the maximum osmotic pressure that is compatible with the minimum magnetic response that is needed for separation in the model system magnetite/poly(sodium acrylate) (Fe_3_O_4_/PAANa). This approach requires the preparation of a range of magnetic nanocomposites (MNC) of magnetic nanoparticles of different sizes and different PAANa/Fe_3_O_4_ ratios, as well as characterizing them and comparing their osmolalities and magnetic properties. Taking into account the need of green chemical procedures that are cheap and easily scalable, we selected two synthesis methods in aqueous solution at low temperatures with an absence of surfactants or organic solvents: the standard coprecipitation method of mixtures of Fe(III) and Fe(II) salts [37], and the oxidative precipitation starting from an Fe(II) salt that is able to produce the highest magnetically responsive magnetite nanoparticles of particle sizes among 20–50 nm [38,39]. Nanocomposites were produced in one step by precipitation of the iron salt in the presence of PAANa, or in a two-step process by subsequent coating of the previously prepared magnetite nanoparticles. The general picture obtained will inform regarding the real capability of FO in water remediation when the recovery of the treated water had to be done exclusively by magnetic forces.

## 2. Materials and Methods

### 2.1. Materials

Iron(III) chloride solution 45%, iron chloride tetrahydrate ≥99%, iron(II) sulfate heptahydrate ≥99%, iron(III) nitrate nonahydrate >99.99%, sodium nitrate ≥99%, sodium hydroxide ≥98%, potassium hydroxide >85%, potassium nitrate >99%, nitric acid >65%, hydrochloric acid >37%, sulfuric acid 0.01 M, ammonium hydroxide solution 28%, poly (sodium acrylate) PAANa Mw of 2100 Da and triethylene glycol (TREG) 99% were bought from Sigma Aldrich (St. Louis, MO, USA) and used as received.

### 2.2. Synthesis of MNC and MNP

Two synthesis methods were used for the production of the MNPs: coprecipitation starting from an Fe(III) and an Fe(II) salt, and oxidative precipitation starting from an Fe(II) salt. Two different approaches were followed for the production of nanocomposites (NC): (1) preparation of pure magnetite MNP and subsequent coating with PAA, and (2) preparation of MNPs in the presence of PAA.

#### 2.2.1. Coprecipitation

In the case of the two-step process, magnetite nanoparticles were obtained by rapidly adding 75 mL of 25% NH_4_OH to 0.5 L of an aqueous solution with 0.175 M of FeCl_3_ and 0.334 M of FeCl_2_. After 5 min of stirring, the MNP were separated by magnetic decantation. In a second step, the surface of the MNP was activated by acid treatment as follows [40]: 300 mL of 2 M of HNO_3_ were added to the decanted MNP and redispersed by stirring; the supernatant was removed after the magnetic sedimentation, and the MNP were redispersed in 75 mL of 1 M Fe(NO_3_)_3_ and 130 mL of water, and the slurry was heated at 90 °C for 30 min. The MNP were recovered again by magnetic decantation, washed with 300 mL of 2 M HNO_3_ once, and then washed three more times with water to remove the excess of acid.

In the case of the one-step process, coprecipitation in the presence of PAANa was carried out by adding different amounts of solid PAANa (2–50 g) to the alkaline precipitant solution containing NH_4_OH, FeCl_3_, and FeCl_2_. The mixing during the precipitation was helped with a high-speed homogenizer Ultra-Turrax^®^ T25, (10,000 rpm, disperser IKA 525N-25G-ST, IKA-Werke GmbH, Staufen, Germany). No acid treatment was performed on these samples. The excess of reagents was removed by extensive dialysis (using a 10,000 Da cut-off membrane), and the product in powder form was obtained by lyophilization.

#### 2.2.2. Oxidative Precipitation

In a glove box under nitrogen, two solutions were prepared using degassed water: (A) 2.12 g NaNO_3_ with 0.7 g NaOH in 225 mL of water; and (B) 1.67 g of FeSO_4_·7H_2_O in 25 mL of H_2_SO_4_ 0.01 M. Solution B was added quickly to solution A while stirring vigorously for 15 min (final concentration of reagents: 0.024 M of FeSO_4_, 0.07 M of NaOH, and 0.1 M of NaNO_3_). The green rust that was initially formed was transferred to a double-jacketed recipient in which up to 50 g of solid PAANa powder was placed beforehand. When the polymer was present, the mixture was homogenized by 1 min of Ultra-Turrax^®^ under the same conditions as in the coprecipitation process. Immediately, the liquid from the thermostat that was set at 90 °C was pumped into the external jacket for fast heating of the slurry without stirring. When 90 °C was reached (15 min), the reactor was closed, and the solution was kept at 90 °C for 24 h. After that, the suspension was cooled at room temperature, the solid was separated by magnetic decantation outside the glove box, and then it was washed several times with distilled water. In the presence of PAANa, acetone addition is needed to promote the separation of the nanocomposite. Finally, a final volume of 50 cc was extensively dialyzed using a 10,000 Da cut-off membrane, and the product in powder form was obtained by lyophilization.

#### 2.2.3. Coating of MNP: A Two-Step Process

MNC can be alternatively obtained by a chemical reaction of PAANa and the bare MNPs at 280 °C as follows [41]: 6 g of dry MNPs were dispersed with sonication for 5 min in 64 mL of TREG and added to a slurry prepared beforehand by Ultra-Turrax^®^ dispersion over 12 h at 90 °C with 6 g of PAANa in 400 mL of TREG (or 800 mL of TREG/water 50% *w*/*w*). The black suspension was overhead stirred at 200 rpm, and heated to 280 °C under nitrogen very slowly for control of the foam formation and a simultaneous distillation of excess of water. Finally, 280 °C was maintained for 30 min under reflux, with a heavy stream of N_2_. After cooling, the mixture was precipitated with acetone, and washed three times with water using magnetic separation. Finally, the suspension was dialyzed and lyophilized.

### 2.3. Characterization of MNC’s

The dry product was characterized by X-ray powder diffraction (XRD) to check the absence of secondary phases. Particle size was evaluated by the use of the Scherrer equation from the width of the XRD (D8 Advance, Bruker, Billerica, MA, USA) peaks (d_XRD_) and from the direct measurements on the TEM micrographs (200 keV JEOL-2000 FXII) (d_TEM_). The presence of PAANa was adsorbed, and the type of linking was assessed by Fourier transform infrared spectroscopy (FTIR) (spectrophotometer IFS 66V-S, Bruker, Billerica, MA, USA), and thermogravimetric analysis (ATD/DSC/TG Q600, TA Instruments, New Castle, DE, USA) was performed in air (up to 800 °C at a scan rate of 10°/min). The amount of PAANa was determined from elemental analysis (EA) of the carbon content (CNHS Elemental Analyzer PERKIN ELMER 2400, Perkin Elmer, Waltham, MA, USA), and the iron content was expressed as magnetite and obtained by a chemical analysis of iron by inductively coupled plasma optical emission spectroscopy (ICP-OES) (Plasma Emission Spectrometer ICP PERKIN ELMER mod. OPTIMA 2100 DV, Perkin Elmer, Waltham, MA, USA). The magnetic properties of the nanocomposites in powder form were determined in a vibrating sample magnetometer (MagLab^®^ VSM Oxford Instruments, Abingdom-on-Thames, UK) at room temperature. The saturation magnetization (Ms) was evaluated by extrapolating the experimental results to an infinite field obtained in the field range where the magnetization increases, which can be described by a 1/*H* law. The magnetic susceptibility was evaluated as the slope of the linear dependence of the magnetization with the applied field at low fields. The hydrodynamic size of the aqueous dispersions was studied by dynamic light scattering (DLS) (Nanosizer^®^ ZS, Malvern, UK), and the osmolality of the dispersions were determined using a freezing point osmometer (model n° 3320 from Advanced Instruments Inc., Norwood, MA, USA).

## 3. Results and Discussion

### 3.1. Characterization of MNCs Prepared by a Two-Step Process

Uncoated MNPs obtained by coprecipitation and oxidative precipitation were characterized in previous works and present mean diameter sizes of 11 nm and 30 nm, respectively [42,43]. Although the polyacrylate coating developed in this work afforded stable aqueous colloids at a pH of around 7, the amount of coating material on the nanoparticles surface was very low: 6% for the 11-nm MNP obtained by coprecipitation, and 1% for the 30-nm MNP obtained by oxidative precipitation. According to that, the osmolalities attained by the dispersions at 33 wt % concentration were 50 mOsm/kg and 10 mOsm/kg, respectively. However, the magnetic response of the samples, which was evaluated here from the saturation magnetization value at room temperature (Ms), was very high: 74 Am^2^/kg for the 11-nm sample, and 80 Am^2^/kg for the 30-nm sample, suggesting that both particles could be easily separated by magnetic fields. Unfortunately, their low osmolalities drastically limit the use of these nanoparticle suspensions in FO processes.

### 3.2. Characterization of MNCs Prepared by a One-Step Process

#### 3.2.1. Samples Prepared by Coprecipitation in Presence of PAA

**Figure 1:**
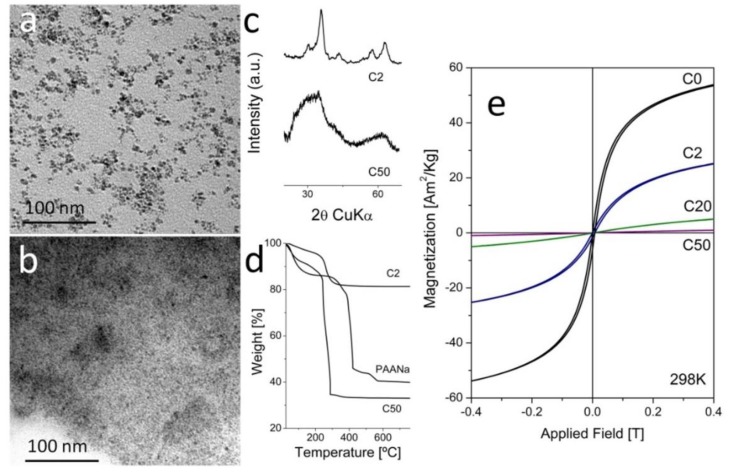
Characterization of magnetic nanocomposites (MNCs) produced by coprecipitation in one step. (**a**,**b**) TEM micrographs of C2 and C50; (**c**) Comparative XRD of samples C2 and C50; (**d**) Comparative TG of samples C2, C50 and pure poly(sodium acrylate(PAANa); (**e**) Magnetization curves at room temperature.

In Figure 1a, we present electron micrographs of two representative samples obtained in the presence of 2 g of PAANa and 50 g of PAANa (samples C2 and C50) with mean particle sizes of 4.4 nm and 2.7 nm (TEM), respectively. The XRD diffraction patterns, chemical analysis, and thermogravimetric curves (Figure 1b,c) confirm that the samples are magnetite/maghemite with Scherrer sizes of 4.4 nm and 3.1 nm, respectively, and with PAANa proportions of 19.2% and 77.0%, respectively. Unfortunately, the magnetic properties of the samples are strongly affected by the PAANa content with an important reduction of the saturation magnetization down to 15–5 Am^2^/kg, which means a reduction of 80% of the magnetization with respect to the particles prepared in the absence of a polymer (71 Am^2^/kg). A superparamagnetic behavior with zero coercivity and remanent magnetization is observed at room temperature for all of the samples, which is in agreement with the small particle size (<5 nm) [44].

#### 3.2.2. Samples Prepared by Oxidative Precipitation in Presence of PAANa

**Figure 2:**
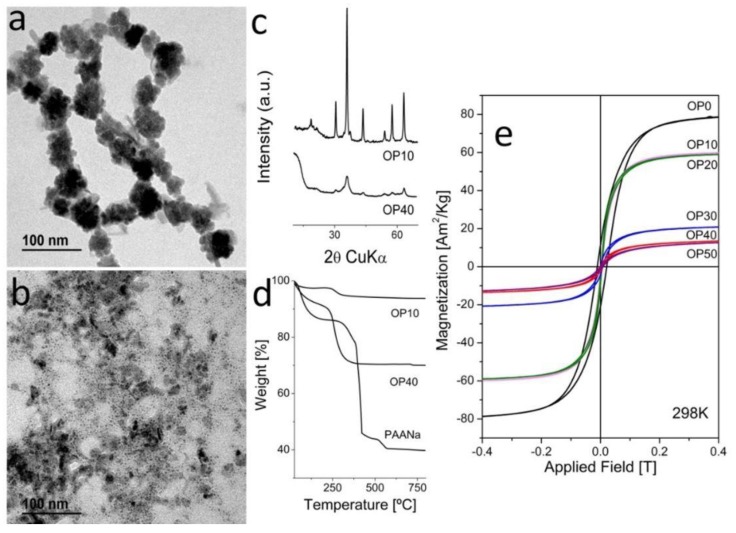
Characterization of MNCs produced by oxidative precipitation in one step. (**a**,**b**) TEM micrograph of OP10 and OP40; (**c**) Comparative XRD of samples of OP10 and OP40; (**d**) Comparative TG of samples of C10, C40, and pure poly(sodium acrylate (PAANa); (**e**) Hysteresis cycles at room temperature.

**Figure 3:**
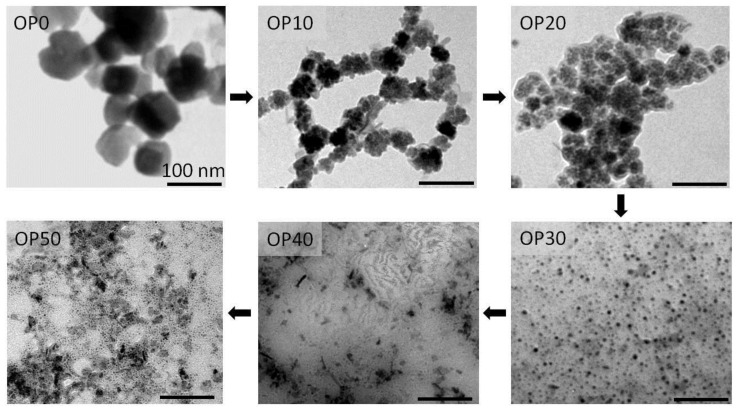
Evolution of the microstructures of MNCs obtained by oxidative precipitation as the proportion of PAANa increases.

In Figure 2a, we present electron micrographs of two representative samples obtained by oxidative precipitation in the presence of 10 g of PAANa and 40 g of PAANa (samples OP10 and OP40) with average particle sizes from around 28 nm to 4 nm (TEM), respectively. The XRD diffraction patterns (Figure 2b), chemical analysis, and thermogravimetric curves (Figure 2c) confirm that the sample is magnetite/maghemite with Scherrer sizes of 34 nm and 5 nm respectively, and with a PAANa proportions of 24.5% and 37.8% respectively. The dramatic change in the nanostructure of the composites with the increase of the proportion of PAANa is remarkable. Up to 20 g of PAANa (mass ratio in the reactant mixture W_PAANa_/W_Fe_ = 59) magnetite nanocrystals appeared in form of aggregates with the polymer among the individuals, but above this limit, magnetite nanocrystals appeared isolated and distributed evenly in the polymeric matrix (Figure 3).

**Figure 4:**
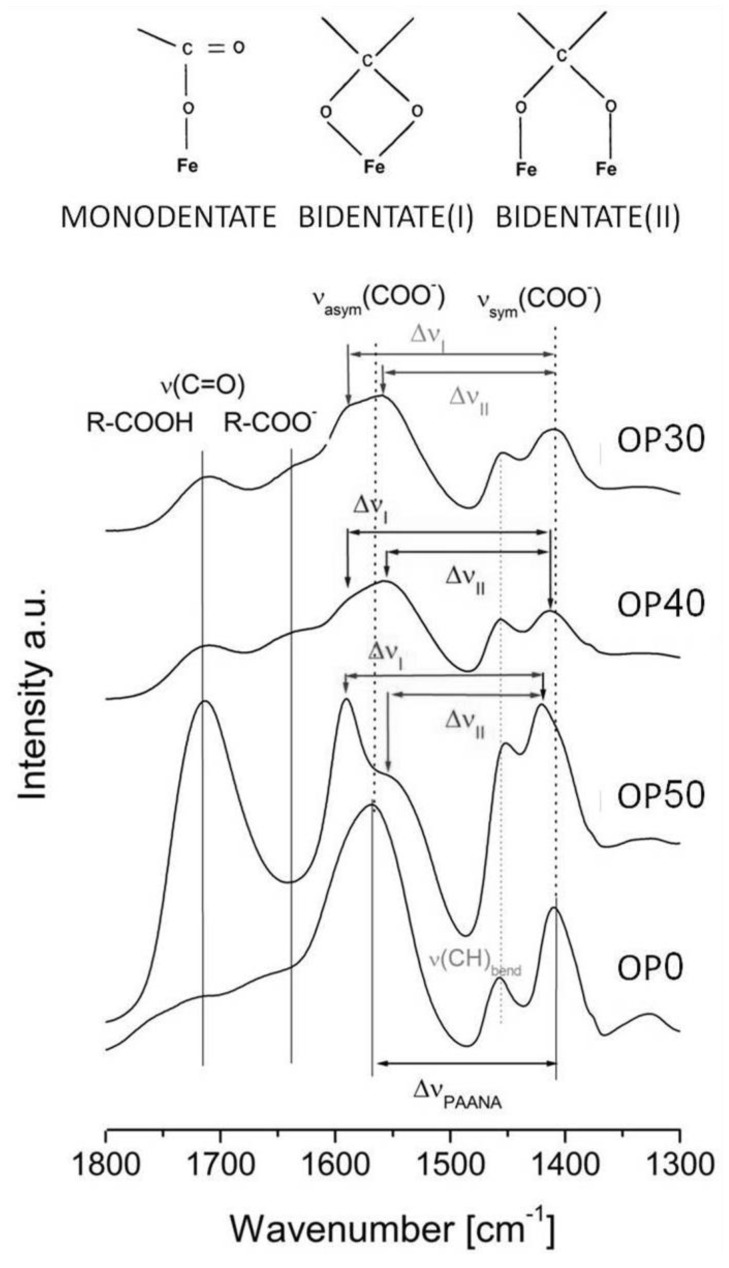
Infrared spectra of the MNCs obtained by oxidative precipitation. The evolution of the value of Δν = ν(COO^−^)_asym_ − ν(COO^−^)_sym_ is presented. Monodentate coordination arises when Δν > Δν_PAANa_ and ν(C=O) exist, bidentate(I) arises when Δν < Δν_PAANa_ and ν(C=O) do not exist, and bidentate(III) arises when Δν ≈ Δν_PAANa_ and ν(C=O) exist.

The interaction among the PAANa and the magnetite nanocrystals were studied by infrared spectroscopy and analyzed using the principles established by Jones, Barrow and Bronswijk [45]. The displacements of the carboxylate bands to lower wavenumbers, the increase of the difference among the symmetric and antisymmetric components with respect to the isolated PAANa, and the predominance of the carbonyl band in high PAANa composites are coherent with monodentate coordination (Figure 4).

As in the case of the samples prepared by coprecipitation, the saturation magnetization decreases strongly with the amount of PAANa from 120 Am^2^/kg down to 19 Am^2^/kg. The results of the characterization of all of the samples produced in this work are summarized in Table 1. Interestingly, when we compare the saturation magnetization of the samples in Am^2^/kg Fe with reference magnetite samples of the same particle size, we obtained good concordance, except for sample OP30 (30 g PAANa), which presented a value that was abnormally low (Figure S1 and Table S1). We hypothesize that in sample OP30, the polymer was somewhat incorporated in the structure of the magnetic material, provoking the internal frustration of the spin interactions. Another indication of the intimate association of the polymer and the magnetite cores comes from the important shift of the decomposition temperature of the PAANa to lower temperatures in the composites with respect to the pure PAANa (Figure 1d and Figure 2d and Figure S2).

On the other hand, the coercivity is reduced to zero when the amount of polymer is higher than 30%, showing superparamagnetic behavior, according to the magnetic particle size reduction and probably the presence of the polymer-reducing interparticle magnetic interactions [44]. However, samples OP0, OP10, and OP20 have some residual coercivity that may result in a higher tendency to aggregation in suspension, and finally a lack of colloidal stability.

#### 3.2.3. Osmolality and Osmotic Pressure as a Function of the Concentration

**Figure 5:**
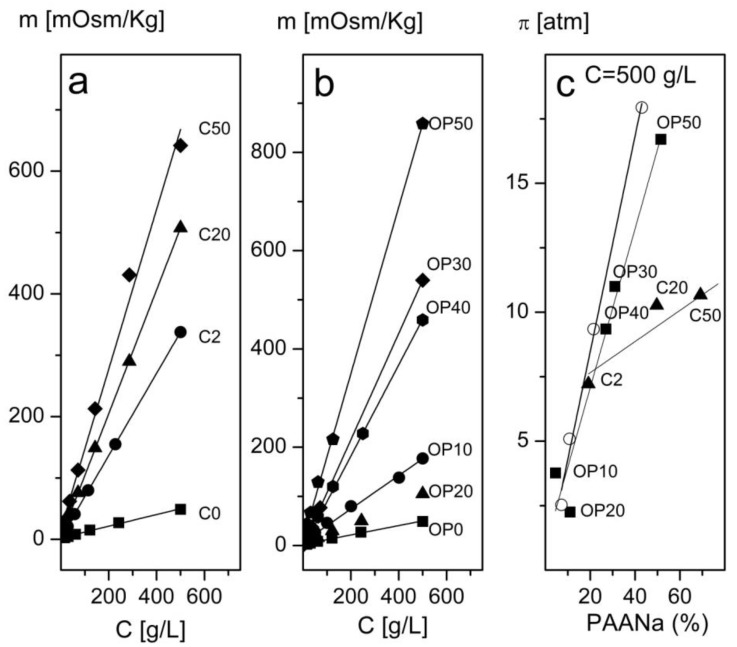
Variation of the osmolality with the concentration for samples of MNCs prepared by coprecipitation (**a**) and oxidative precipitation (**b**) in one step measured at room temperature and pH = 8. In (**c**), we present the evolution of the osmotic pressure with the proportion of PAANa for all of the samples, the results obtained by equivalent aqueous solutions of pure PAANa are presented for comparison (open circles).

The values of the osmolalities measured as a function of the mass concentration up to the maximum practical concentration considering FO experiments (500 g/L) and at a pH = 8 (the maximum operative pH of the HTI OsMem^®^ separation membranes (Hydration Technology Innovations LLC, Albany, OR, USA)) to maximize the proportion of ionized –COO^−^ groups in PAANa are presented in Figure 5a,b, where it can be observed that the osmolalities increased linearly with the concentration for both sets of samples with slopes dependent on the PAANa content in the composite. The osmotic pressure is computed using the van’t Hoff expression (Equation (1)).

(1) π=cRT,

where R = 0.082 L·atm·K^−1^·Mol^−1^, c was obtained from the experimental osmolality (m) that was measured with the freezing point osmometer and converted to molarities (c) using the expression (Equation (2)), and d and C are the experimental density and mass concentration of the dispersion in g/cc, respectively.

(2) c=m(d−C),

In Figure 5c, we present the evolution of the osmotic pressure with the PAANa concentration in the dispersion at the maximum concentration of composite (500 g/L) for both sets of samples and the correspondent to the pure PAANa for comparison. The osmotic pressure values attained in the Fe_3_O_4_/PAANa composites obtained by oxidative precipitation were similar to the ones produced by the PAANa alone, but those prepared by coprecipitation show a different trend that led to lower osmotic pressures than expected for their PAANa content. This could be due to differences in the conformation of the polymer, starting from the known tendency of polyelectrolytes to lay planar when adsorbed on polar surfaces [46]. We hypothesize that the PAANa that adsorbed during the coprecipitation of magnetite followed this trend, and the onion-like structure formed generate smaller osmotic pressures than the free polymer. Whereas, perhaps due to the higher temperature of the synthesis of the oxidative precipitation, the PAANa that was adsorbed destabilized the planar configuration in favor of more open configurations (which were more similar than those adopted by free PAANa in solution) was more effective in the interaction with water.

#### 3.2.4. Magnetic Response

The magnetic response of a colloidal system formed by magnetic nanoparticles, in the concentration range that is needed for its use as draw electrolyte, is difficult to predict due to the magnetic interactions that tend to reduce the effective field with respect to the input field [47] and the effect of the aggregation that favors cooperation among the magnetic cores. Particles with a sufficient high moment may form clusters with different configurations such as chains, loops, or anisometric aggregates that will modify its magnetic response, but these will not be considered here. In any case, the magnetic energy (E) and the magnetic force (F) are given by the expressions in Equations (3) and (4) [48]:

(3) $${E=\mu }_{0}\mathrm{mH},$$

(4) $${F=\mu }_{0}\mathrm{mH}\nabla H,$$

where μ_0_ = 1.2566 × 10^−6^ VsA^−1^m^−1^ is the permeability of free space, m is the magnetization of the colloidal aggregate of Fe_2_O_3_/PAA nanoparticles in the aqueous dispersion, and H is the magnetic field. The key parameter is the maximum magnetization that is attained by the aggregate, which will depend on the aggregate size and the saturation magnetization. The aggregate size of the colloids is controlled by the amount of PAANa that is present in the system irrespective of the method of synthesis. The control is made through the effect on particle size, but when present, the proportion of PAANa in the aggregate does not affect the hydrodynamic size too much (Table 1, Figure S3). The latter is in the range of 150–200 nm, which assures stability at neutral pH, but reduces the density of magnetic cores in the aggregates with high PAANa content. In conclusion, the key parameter to characterize the magnetic response of the composites is the saturation magnetization (m_s_) of a single MNP coated with PAANa (Equation (5)).

**Figure 6:**
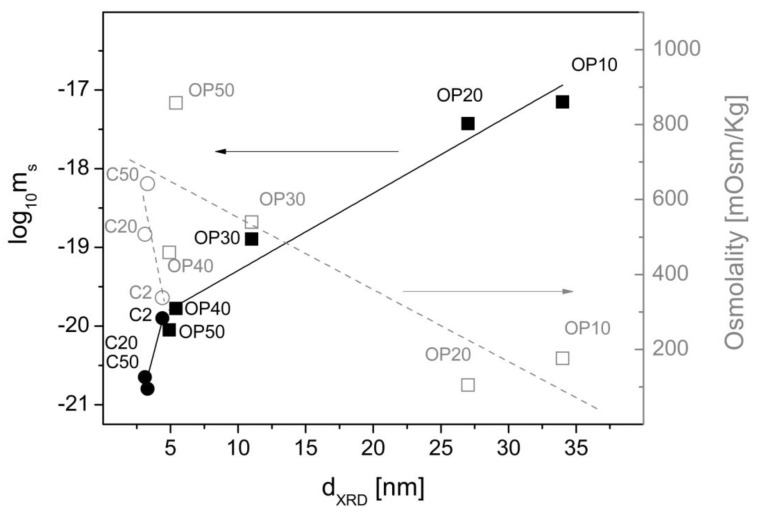
Dependence of the magnetic response and the osmolality of MNCs as a function of the particle size obtained from the XRD data. Full square points (■) represent the magnetic response of samples produced by oxidative precipitation. Full round points (●) represent the magnetic response of samples produced by coprecipitation. Empty square points (□) represent the osmolality for samples produced by oxidative precipitation, and empty round points (○) represent the osmolality for samples produced by coprecipitation, in both cases at 500 g/L.

(5) ms=43π(dXRD·10−92)3dFe3O4·(1+PAANa100−PAANa)·Ms,

where d_Fe3O4_ is the density of the MNP approximated to the magnetite value of 5200 kg/m^3^. Figure 6 presents the logarithm of m_s_ as a function of the particle size. From the linear dependence obtained (with the exception of the smaller nanoparticles that give smaller response due to surface spin canting [44]), it can be seen that the magnetic response drops logarithmically with the reduction of particle size, independently of the method of synthesis and the proportion of PAANa.

The osmolality that was also displayed in Figure 6 shows much higher values for the smaller nanoparticles due to the higher proportion of PAANa, but with much more scattering depending on the synthesis conditions. The dependence on the particle size seems to be approximately linear with a steeper decay for the coprecipitation method.

In general terms, the results of the osmotic pressures and magnetizations presented compare well with previous studies using the same nanocomposites obtained by other methods [49,50]. The advantages of the application of oxidative precipitation for the preparation of the draw solutes presented here come from the simplicity of their preparation, the scalability of the process, and the possibility of obtaining a range of osmolalities and magnetic responses by simply changing the proportion of PAANa in the reaction media.

## 4. Conclusions

The main conclusion attained in this work is that in order to obtain high magnetic response and high osmolality in nanocomposites based on magnetite nanoparticles and PAANa, one-step synthesis is the best strategy. The multistep procedure could successfully functionalize highly responsive magnetic nanoparticles with low coating densities, but these were insufficient to reach high osmotic pressure in a scalable cost-effective way. The single-pot procedure imposes the presence of the polymer on the nanoparticle surface that is responsible for the osmotic pressure in such an amount that impedes the growth of the magnetic cores, which is to the detriment of the magnetic performance. This limitation imposes the need to adjust the experimental conditions of synthesis in order to achieve a compromise for both properties for the particular application envisaged. In this work, we found that the best compromise solution for the system Fe_3_O_4_/PAANa was the nanocomposite OP30, which presents at the same time good magnetic response, >25 Am^2^/kg, superparamagnetic and therefore reversible behavior, and a relatively high osmotic pressure of 11 bar at high concentration. Such draw electrolytes, provided that the PAANa could resist the harsh conditions of repetitive FO processes, could be useful in certain FO applications dealing with polluted continental or wastewater. 

## Figures and Tables

**Table 1:** Results of the characterization of the MNCs obtained by coprecipitation and oxidative precipitation in one step (d_TEM_ = mean diameter by TEM; d_XRD_ = mean crystal size by XRD; D_HYD_ = hydrodynamic size in intensity (Z_ave_) by DLS; Ms = saturation magnetization; χ = initial magnetic susceptibility).

Sample	d_TEM_ (nm)	d_XRD_ (nm)	D_HYD_ (Z_ave_) (nm)	Fe_3_O_4_ ICP wt %	PAANa EA wt %	Ms 298 K Am^2^/kg	Χ 298 K Am^2^/kgT
**C0**	10 ± 2	7.5	170	98	0	71	758
**C2**	4 ± 1	4.4	95	74.0	19.2	43.4	192
**C20**	3 ± 0.8	3.1	170	50.7	49.7	16.7	19
**C50**	3 ± 1	3.3	170	21.7	77.0	5.6	4
**OP0**	50 ± 20	67.3	560	98.7	0	82.5	*
**OP10**	28 ± 5	34.2	270	72.9	4.5	63	1700
**OP20**	32 ± 10	27.5	140	75.5	11.0	62	11,900
**OP30**	7 ± 1	10.9	150	53.0	31.0	24	463
**OP40**	4 ± 1	4.9	220	52.6	27.0	20	219
**OP50**	3 ± 0.6	5.4	170	41.1	51.5	19	186

(*) The sample OP0 is not superparamagnetic, and the magnetic susceptibility is not defined.

## Supplementary Materials

The following are available online at http://www.mdpi.com/2079-4991/8/7/470/s1, Figure S1: Hysteresis cycles of Fe_3_O_4_/PAANa nanocomposites obtained by oxidative precipitation expressed in Am^2^/Kg Fe, Figure S2: Thermogravimetric analysis of all samples showing the shift in the decomposition temperature of adsorbed PAANa. Figure S3: 3D Plot of the dependence of the hydrodynamic size on the magnetic particle size (d_XRD_) and PAANa wt % content. Table S1: Comparison of the saturation magnetization per Kg of iron present in the composite samples of Fe_3_O_4_/PAANa obtained by oxidative precipitation with the reference samples of pure magnetite/maghemite of similar size.
