# Supplementary material for: Effect of the Sodium Polyacrylate on the Magnetite Nanoparticles Produced by Green Chemistry Routes: Applicability in Forward Osmosis
Source: Nanomaterials (Basel). 2018 Jun 27;8(7):470. doi: 10.3390/nano8070470 (PMC6071008; doi:10.3390/nano8070470)
Supplement: Supplementary file 1 [file nanomaterials-08-00470-s001.pdf]

nanomaterials

Effect of the Sodium Polyacrylate on the Magnetite Nanoparticles Produced by Green Chemistry Routes: Applicability in Forward Osmosis

Juan Zufía-Rivas, Puerto Morales and Sabino Veintemillas-Verdaguer *

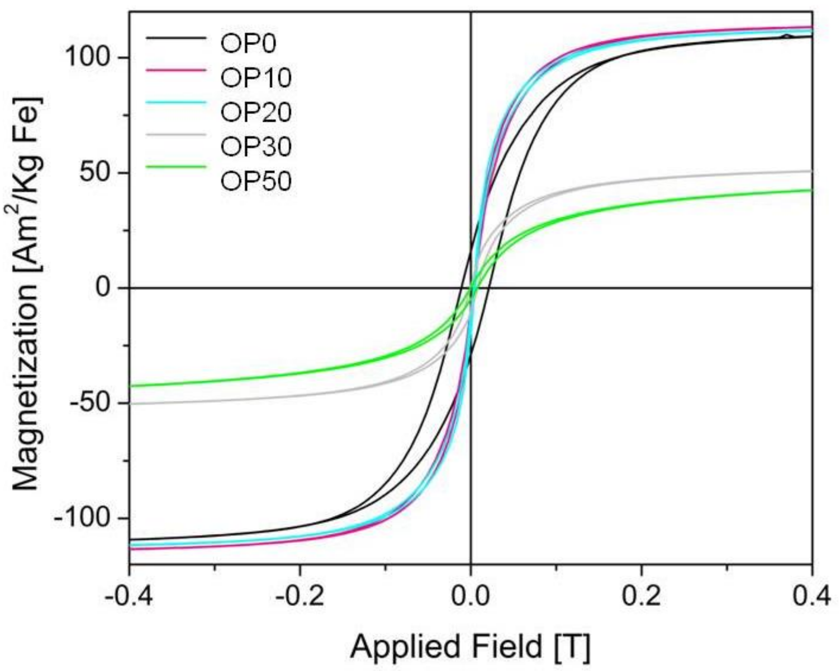

Figure 1S. Hysteresis cycles of Fe_3O_4/PAANa nanocomposites obtained by oxidative precipitation expressed in Am^2/Kg Fe.

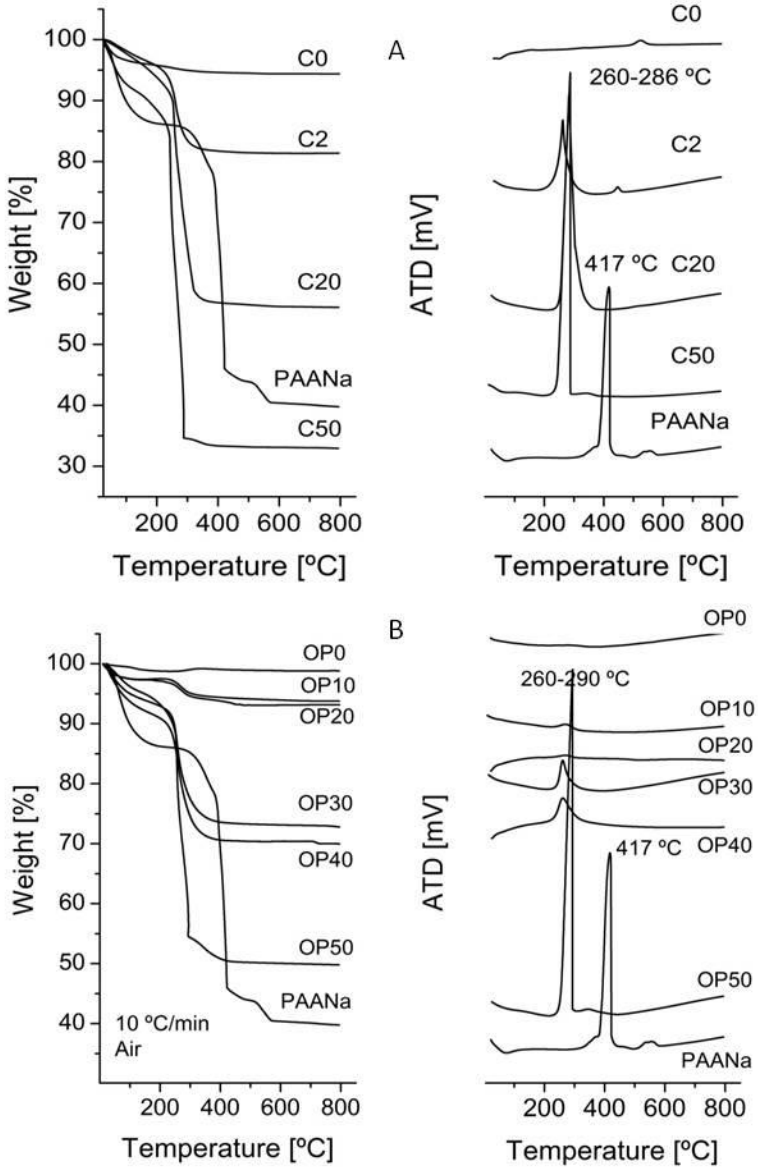

Figure 2S. Thermogravimetric analysis of all samples showing the shift in the decomposition temperature of adsorbed PAANa.

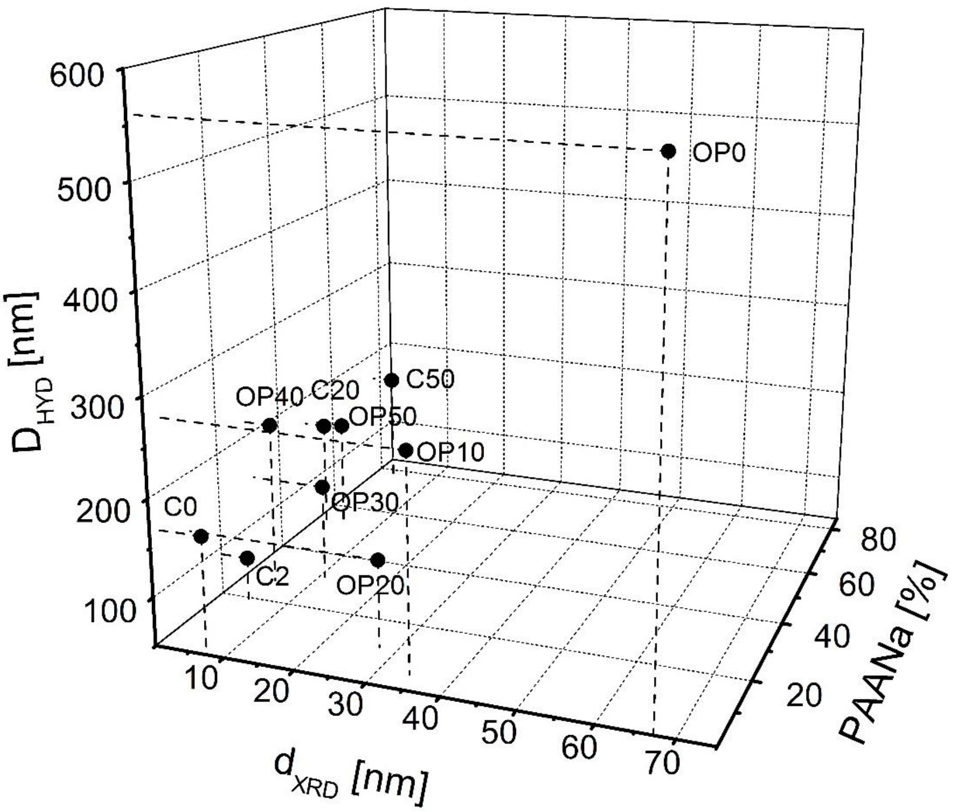

Figure 3S. 3D Plot of the dependence of the hydrodynamic size on the magnetic particle size d_{XRD} and PAANa wt% content .

TABLE 1S

Comparison of the saturation magnetization per Kg of iron present in the composite samples of Fe_3O_4/PAANa obtained by oxidative precipitation with the reference samples of pure magnetite/maghemite of similar size

298 K	**$D_{SHERRER}$ nm**	**Ms (Fe) Am²/KgFe**	**γ (Fe) Am²/KgFeT**	**Pure samples as reference**	
				**Size [nm]**	**Ms(Fe) Am²/KgFe**
OP10	34	120.0 ± 0.4	3170 ± 178	*35*	120
OP20	27	117.8 ± 0.3	3094 ± 157	*25*	114
OP30	11	**63.8** ± 0.2	1205 ± 197	*10*	**86**
OP50	5	63.8 ± 0.2	636 ± 70	*5*	56
